# More Honours

**Published:** 1842-12

**Authors:** 


*More Honours.*—In a later number of Galignani's Messenger, we learn that Dr. Brewster had presented to him by Prince Louis Napoleon, a splendid gold snuff-box, with the likeness of the emperor upon it, as a testimony of the value he placed upon his professional services while on a visit to Hamburg.

